# Tuina therapy plus resistance exercise vs. Tuina alone for mechanical neck pain: a randomized controlled trial

**DOI:** 10.3389/fmed.2026.1709232

**Published:** 2026-01-19

**Authors:** San Zheng, Zhiwei Wu, Yaping Chang, Hua Xing, Yiming Shan, Yangyang Fu, Yazhou Li, Zhiran Kang, Jintian Chen, Jiangshan Li, Jie Li, Junliang Wang, Min Fang, Wuquan Sun

**Affiliations:** 1 Department of Tuina, Yueyang Hospital of Integrated Traditional Chinese and Western Medicine, Shanghai University of Traditional Chinese Medicine, Shanghai, China; 2 Institute of Tuina, Shanghai Institute of Traditional Chinese Medicine, Shanghai, China; 3 Department of Health Research Methods, Evidence & Impact (HEI), Faculty of Health Sciences, McMaster University, Hamilton, ON, Canada; 4 Department of Tuina, Shuguang Hospital Affiliated to Shanghai University of Traditional Chinese Medicine, Shanghai, China

**Keywords:** complementary and alternative medicine, isometric contraction training, mechanical neck pain, resistance exercise, Tuina therapy

## Abstract

**Background:**

Tuina and exercise therapy are widely used to treat mechanical neck pain (MNP), but evidence on their combined efficacy remains limited. This study evaluated the effectiveness of Tuina combined with resistance exercise (RE) versus Tuina alone in managing MNP.

**Methods:**

We conducted a 4-week, analyst-blinded, randomized controlled trial with 90 participants with MNP. Participants were randomly assigned to receive either Tuina Therapy plus RE (TTRE, *n* = 45) or Tuina alone (*n* = 45). Both groups underwent two Tuina sessions per week for 4 weeks (eight sessions in total). In addition, the TTRE group performed RE three times daily for 4 weeks. Each RE consisted of 5 s of static resistance followed by 2 s of relaxation. The number of repetitions per session increased progressively: 5 in week 1, 10 in week 2, 15 in week 3, and 20 in week 4. The primary outcome was the change in pain visual analog scale (VAS) score from baseline to week 4. Secondary outcomes included the Neck Disability Index (NDI) score, peak strength of cervical muscle (PSCM), cervical range of motion (CROM), cervical curvature (Cobb Angle), and adverse events.

**Results:**

The mean age of the 90 enrolled patients was 26.4 years [standard deviation (SD), 3.1 and 49 (54.4%) were female]. The mean difference in VAS scores from baseline at week 4 for TTRE group was −4.2 (95% CI, −4.4 to −4.0). At week 4, the difference in VAS score was 0.5(95% CI, 0.30 to 0.77; *p* < 0.001) between Tuina group and TTRE group.

**Conclusion:**

In this study, participants with MNP in the TTRE group showed statistically greater improvements than those in the Tuina group in pain reduction, functional recovery, extension PSCM, and flexion CROM at week 4. TTRE may be considered a valuable option in the management of MNP.

**Clinical trial registration:**

We registered the trial with the Chinese Clinical Trial Registry (ChiCTR2300068344; Registration Date: February 15, 2023) at http://www.chictr.org.cn.

## 1 Background

Mechanical neck pain (MNP) refers to pain localized in the cervical spine, occipital region, or posterior scapular area, typically accompanied by restricted neck mobility (1). This musculoskeletal disorder has an annual prevalence exceeding 30% and may affect up to 50% of the general population at some stage in their lives (2). As the fourth leading cause of disability worldwide, neck pain can profoundly impair physical function, psychological health, and social well-being, and it places a considerable burden on healthcare systems through increased costs (3). The prognosis of MNP is influenced by multiple factors, including the duration of symptoms, the presence of associated clinical features, and the nature and timing of therapeutic intervention (4). In the absence of appropriate management, symptoms may persist or progressively deteriorate into chronicity, as the potential for spontaneous resolution is generally limited (5). Evidence from clinical studies indicates that control groups typically exhibit only minimal improvements in pain intensity and neck-related functional outcomes over short-term durations, such as 4 weeks (6, 7). These findings underscore the limited potential for spontaneous recovery within this timeframe and reinforce the imperative for active therapeutic intervention. Various conservative therapies are recommended for the treatment of MNP (8, 9). For example, nonsteroidal anti-inflammatory drugs (NSAIDs) are frequently employed in the management of MNP due to their established efficacy and remain widely used; however, their widespread application continues to raise concerns regarding their adverse event profile, necessitating careful monitoring and individualized consideration (10, 11). Chiropractic care has been widely utilized in healthcare systems for managing neck and back pain as a non-invasive approach to alleviate symptoms and improve function (12, 13). Consequently, non-pharmaceutical therapies, such as traditional Chinese medicine and therapeutic exercise, warrant increased consideration as viable alternatives.

Traditional Chinese manual therapy, known as Tuina in China, has been practiced for thousands of years. Tuina typically involves stimulation of specific acupuncture points along meridian pathways and incorporates passive mobilization techniques targeting the patient’s joints. The therapeutic effects are mediated through a range of manual techniques (e.g., pressing, pushing, kneading), characterized by the sustained, controlled, and precise application of force that is simultaneously deep and gentle (14). The efficacy and safety of Tuina in pain management have been substantiated in clinical practice, particularly in the treatment of neck pain (15), low back pain (16, 17), and knee pain (18, 19). Our previous experimental studies suggest that the analgesic effects of Tuina are mediated through modulation of neuroimmune pathways (20, 21), suppression of microglial activation (22), regulation of inflammatory mediators (23), and induction of cortical remodeling (24). Tuina may also attenuate intervertebral disk degeneration by mitigating oxidative stress and modulating enzymatic activity (25). Despite these benefits, the effectiveness of Tuina appears to be constrained by the limited frequency of treatment sessions (26). Given that Tuina is inherently passive and practitioner-dependent, its integration with active, home-based exercise interventions may enhance long-term therapeutic outcomes.

Among complementary interventions for neck pain, exercise therapy possesses the most robust evidence base (27). Resistance exercise (RE), in particular, is practical, widely accessible, and effective in enhancing muscle strength (28). A systematic review further demonstrated that RE yields greater reductions in neck pain and disability compared with alternative exercise modalities (29). Moreover, electromyographic studies in patients with patellar tendinopathy suggest that isometric RE may alleviate cortical inhibition and facilitate motor cortical reorganization, mechanisms that may contribute to its analgesic effects (30, 31). Despite its clinical utility, the evidence base for RE in MNP remains limited, primarily due to a paucity of high-quality randomized controlled trials (RCTs). This gap underscores the need for rigorously designed protocols that systematically account for critical variables such as exercise intensity, frequency, and recovery intervals (32). Recent investigations into the dose–response relationship of RE indicate that a single daily 10-min high-intensity session may provide pain relief comparable to that achieved with two 10-min sessions over a 16-week intervention period (33). Notably, evidence from other musculoskeletal pain conditions has suggested potential advantages of combining RE with manual therapy. A recent RCT in knee osteoarthritis patients reported superior pain outcomes with a RE-based combined intervention compared with a single-modality approach (34). Although these findings cannot be directly extrapolated to neck pain, they support the broader rationale that multimodal strategies may better address the complex and multifactorial nature of musculoskeletal pain, reinforcing the increasing clinical interest in combination therapy (35, 36).

Therefore, this study aimed to evaluate the efficacy of Tuina therapy plus RE (TTRE) in the management of MNP. Given the substantial societal and familial economic burden associated with MNP and related pain conditions—reflected in an annual healthcare expenditure of approximately NOK 6.35 billion in Norway [2019; (37)]—together with the scarcity of high-quality RE RCTs (29), this trial hypothesized that TTRE would be superior to Tuina alone in reducing pain intensity. The primary outcome was the change in pain intensity, as measured by the visual analog scale (VAS) scores, following a 4-week intervention. We further hypothesized that TTRE would lead to greater improvements in disability and muscle strength compared with Tuina alone.

## 2 Methods

### 2.1 Study design

**Figure 1:**
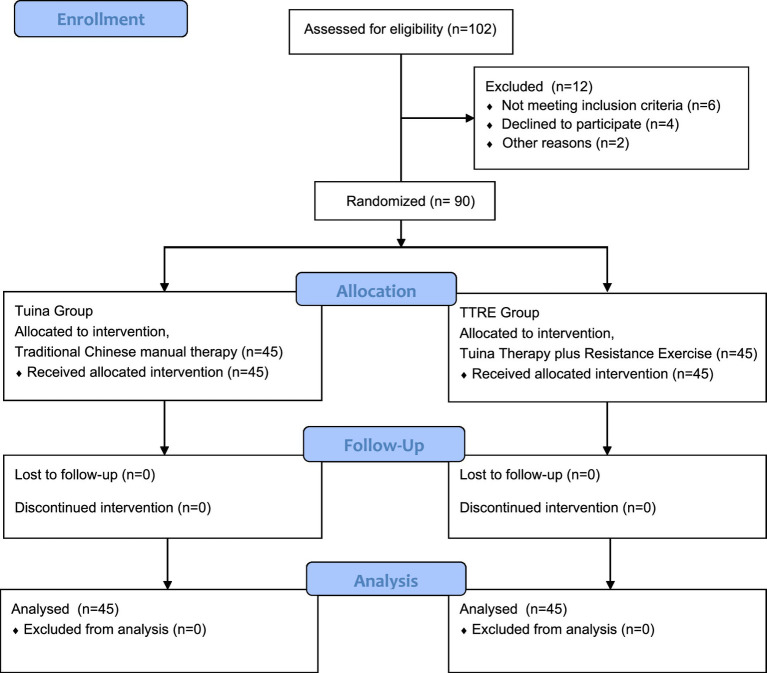
Flowchart of study design.

We conducted a 4-week RCT with two parallel arms: a control group receiving Tuina and an intervention group receiving TTRE. The study protocol was approved by the regional ethics review committee of Yueyang Hospital of Integrated Traditional Chinese and Western Medicine. We determined the study sample size based on the primary pain VAS outcome. According to data from a previous study (35), the expected post-treatment VAS scores were 3.67 for patients with MNP receiving Tuina therapy and 2.87 for those receiving TTRE. Sample size estimation was performed using the Giga Calculator, assuming a statistical power of 80%, a two-sided significance level of 5%, and a 95% confidence interval. The required sample size was estimated to be 82 participants. To account for an anticipated attrition rate of approximately 10%, the final target sample size was increased to 90 participants. Accordingly, 90 eligible patients with MNP were randomly assigned to the two groups in a 1:1 allocation ratio. Participant flow, reported in accordance with CONSORT guidelines, is presented in Figure 1. All interventions were administered at Yueyang hospital.

### 2.2 Eligibility criteria

The study enrolled participants of both sexes, aged 18 to 40 years, presenting with neck pain or dysfunction attributed to MNP. Diagnosis was established based on medical history (38), physical examination (39), and radiographic assessment, in accordance with the Canadian C-Spine Rule and the National Emergency X-Radiography Utilization Study (NEXUS) (40).

Exclusion criteria comprised a history of fractures, paraplegia, acute traumatic conditions with uncertain diagnosis, or severe osteoporosis; prior whiplash, head, or neck trauma; neck pain associated with serious systemic diseases (cardiac, cerebral, hepatic, pulmonary, renal, hematologic, or endocrine); women who were pregnant, planning pregnancy, lactating, or postpartum; and individuals intolerant to Tuina or who had participated in other clinical trials within 2 weeks prior to enrollment. Participants were considered withdrawn if they discontinued treatment due to lack of efficacy or adverse effects, lost contact, or were excluded by investigators due to poor compliance or serious adverse events.

### 2.3 Randomization, allocation concealment, and blinding

Participants were primarily recruited from Yueyang hospital. Interested individuals contacted the research team via phone or a WeChat QR code, both of them being the widely used platform for accessing health information in China (41). A researcher verbally explained the study protocol and eligibility criteria, and preliminary eligibility was assessed by telephone following verbal consent. Eligible candidates were then provided with study documents via WeChat or email and were given a minimum of 24 h to review the materials prior to providing consent. Participants who met the eligibility criteria were invited to attend a baseline assessment session, during which eligibility was reconfirmed and written informed consent was obtained. An independent statistician generated the allocation sequence using a random number generator in SPSS version 25.0 (SPSS Inc., Chicago, IL, USA), employing a block size of 10. Allocation concealment was maintained using sequentially numbered, opaque, sealed envelopes prepared by independent staff. Each participant was assigned a unique study ID, and the intervention was revealed upon opening the corresponding envelope. Owing to the nature of the intervention, Tuina practitioners, physical therapists, and participants were aware of group allocation, whereas outcome assessors, data collectors and analysts remained blinded.

### 2.4 Interventions

Patients in both the Tuina and TTRE groups underwent two Tuina sessions per week for 4 weeks. All treatments were delivered by a licensed senior therapist with over 10 years of clinical experience. The intensity of Tuina was individualized based on physical examination, clinical judgment, and participant feedback. The therapist followed a standardized five-step protocol, including Yi Zhi Chan pushing, rolling combined with passive movements, pull-stretching and subtle adjusting cervical spine, pressing Tianzong (SI11) and grasping Jianjing (GB21). The Tuina protocol has been previously reported in our earlier publication (42). A detailed description is provided in the Appendix in Supplementary File 1, and a video demonstration of the specific manual techniques is available in Supplementary Video 1.

In addition to the 8 Tuina sessions, participants in the TTRE group performed a RE protocol commonly used in clinical practice in China (43). The protocol primarily comprised isometric exercises targeting the neck extensor muscles (42). Each repetition lasted 7 s, alternating between exertion and rest. Participants initially performed 5 repetitions per set, three times daily (morning, noon, and evening) for 6 times a week. Repetitions were gradually increased by 5 per set each week, reaching 20 per set by week four. A moderate intensity was considered appropriate, as higher frequency was deemed unnecessary. Each participant received a brief instructional leaflet illustrating proper exercise techniques. To promote adherence and accurate performance, participants were encouraged to record their exercises and share the videos with the therapist via WeChat for review and feedback. Participants were enrolled in a dedicated study group and instructed to submit a complete video of each daily exercise session via an embedded mini-program (Chain Sign-Up Assistant), followed by mandatory group check-in confirmation. Two trained administrators oversaw adherence: one monitored quantitative compliance (completed/prescribed sessions × 100%) and issued reminders for missed sessions, while the other reviewed videos to provide real-time individualized postural corrections and exercise guidance. This system ensured both adherence verification and intervention fidelity. Detailed instructions are provided in the Supplementary File 2.

### 2.5 Outcomes

The primary outcome was the change in pain VAS from baseline to week 4. Secondary outcomes included: (1) VAS at week 1, 2, and 3; (2) Neck Disability Index (NDI) at week 1–4; (3) peak cervical muscle strength (PSCM) at week 4; (4) cervical range of motion (CROM) at week 4; and (5) cervical curvature (Cobb angle) at week 4.

#### 2.5.1 VAS

Pain intensity is assessed using a 10-cm horizontal VAS with 0 representing no pain and 10 the worst imaginable pain (44). The VAS is a reliable and valid measure of pain, with reported intraclass correlation coefficients ranging from 0.96 to 0.98 (45).

#### 2.5.2 NDI

Functional capacity and physical activity were assessed by NDI (46). The questionnaire concludes a total of 10 questions, with each question providing 6 possible answers ranging from 0 (no disability) to 5 (complete disability). The total NDI score ranges from 0 to 50.

#### 2.5.3 PSCM

The PSCM is measured using a Hoggan MicroFET3 dynamometer (Hoggan Scientific LLC, United States), a method shown to be reliable and reproducible for assessing muscle performance (47–49). Four measurements are taken for each neck movement (flexion, extension, left and right lateral flexion), and the average value is recorded.

#### 2.5.4 CROM

The CROM is assessed with the Hoggan MicroFET3 clinometer (Hoggan Scientific LLC, United States). Flexion, extension, and lateral flexion were measured in the seated position, and rotation in the supine position (50). Each movement was measured three times, and the average value was used for analysis.

#### 2.5.5 Cobb angle

Cervical curvature was assessed from lateral radiographs acquired using the radiographic system (Winning Health, WiNEX). The Cobb angle from C2 to C7 was measured independently by two reviewers, a method with high reliability (51). During radiography, the participant was positioned sideways with the chin raised to align the nose line perpendicular to the trunk. The shoulder blades are lowered and retracted toward the midline to optimize imaging.

#### 2.5.6 Adverse events

Any adverse event, such as unfavorable or unintended signs, symptoms, or diseases, related to the Tuina therapy or RE was reported by patients and Tuina doctors. Severe adverse events had to be reported to the principal investigator and the data and safety monitoring board within 24 h after their occurrence.

### 2.6 Statistical analysis

Statistical analyses were performed in accordance with the intention-to-treat (ITT) principle using SPSS version 25.0 (SPSS Inc., Chicago, IL, United States), with missing data addressed by single imputation using the last observation carried forward (LOCF) method. Continuous variables were expressed as mean ± standard deviation if normally distributed, or as median with interquartile range if non-normally distributed. Categorical variables were presented as counts and percentages. Normality was assessed using the Kolmogorov–Smirnov test with Lilliefors correction. Parametric or non-parametric methods were applied according to the results of normality and homogeneity tests. Repeated-measures ANOVA was used to evaluate treatment, time, and their interaction, while paired t-tests assessed within-group changes. Categorical outcomes, including adverse events, were compared using chi-square or Fisher’s exact tests. Statistical significance was defined as a two-tailed *p* < 0.05.

## 3 Results

### 3.1 Participant characteristics

Of 106 individuals screened, 90 patients (84.9%) with MNP met the inclusion criteria and were randomized to either the Tuina group (*n* = 45) or the TTRE group (*n* = 45). The mean [standard deviation (SD)] age was 26.4 (3.1) years, with 49 females (54.4%) and 41 males (45.6%). Baseline characteristics are summarized in Table 1. All participants completed the 4-week outcome assessments. The compliance rate of the RE at week 1, week 2, week 3 and week 4 was 100.0, 88.9, 93.3 and 97.8%, respectively, in the TTRE group, while the compliance rates of Tuina during the 4-week in both groups were high, 100%.

**Table 1:** Baseline characteristics of participants (*n* = 90).

Characteristic	Intervention (*n* = 45)	Control (*n* = 45)
Age, mean (SD), year	26.2(2.5)	26.5(3.6)
Sex, *n* (%)		
Male	23(51.1)	18(40.0)
Female	22(48.9)	27(60.0)
Height, mean (SD), cm	168.6(9.3)	169.0(8.1)
Weight, mean (SD), kg	64.9(14.2)	59.6(8.4)
Duration time for neck pain, n (%)		
<3 months	0(0)	0(0)
> = 3 months to <6	31(68.9)	29(64.4)
> = 6 months to <12	8(17.8)	12(26.7)
> = 12 months	6(13.3)	4(8.9)

CROM, cervical range of motion; IQR, inter-quartile range; NDI, neck disability index; PSCM, peak strength of cervical muscles; SD, standard deviation; VAS, visual analog scale.

### 3.2 Efficacy

Changes in VAS scores from baseline to week 4, the primary outcome, was summarized in Table 2. The Tuina group showed a mean reduction of −4.2 (95% CI, −4.4 to −4.0), while the TTRE group showed a mean reduction of −4.7 (95% CI, −5.0 to −4.4). The between-group difference in VAS change at week 4 was significant in favor of the TTRE group (−0.5; 95% CI, −0.77 to −0.30; *p* < 0.001).

**Table 2:** Primary outcome by group in mechanical neck pain.

Primary outcome	Intervention (*n* = 45)		Control (*n* = 45)		Intervention effect	
	Median (IQR)	Mean change from baseline (95% Cl)	Median (IQR)	Mean change from baseline (95% Cl)	Difference between group (95% Cl)	*p-*value
VAS score (0–10)						
Baseline	6(5 to 7)		6(5 to 7)			
1 wk	6(5 to 6)	−0.4(−0.6 to −0.3)	5(4 to 6)	−0.6(−0.9 to −0.5)	−0.2(−0.46 to 0.02)	0.067
2 wk	4(4 to 5)	−1.6(−1.9 to −1.3)	4(4 to 5)	−1.5(−1.8 to −1.2)	0.2(−0.04 to 0.44)	0.107
3 wk	2(2 to 3)	−3.6(−3.9 to −3.3)	2(2 to 3)	−3.4(−3.6 to −3.1)	0.3(0.02 to 0.52)	**0.038** ^*^
4 wk	1(1 to 2)	−4.7(−5.0 to −4.4)	2(1 to 2)	−4.2(−4.4 to −4.0)	0.5(0.30 to 0.77)	**<0.001** ^*^

Significant *p*-values are in bold font.

IQR, inter-quartile range; VAS, visual analog scale; 95% Cl, 95% confidence interval.

*Indicates a statistically significant difference (*p* < 0.05).

**Figure 2:**
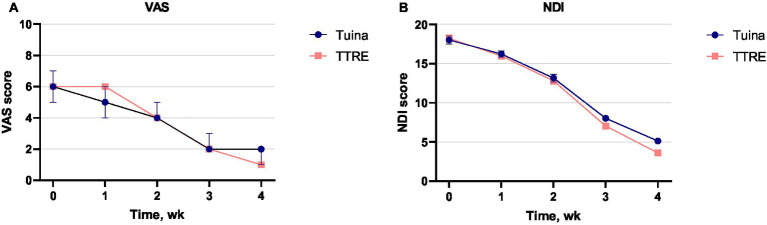
Changes in VAS and NDI scores. **(A, B)** depict the longitudinal changes in visual analog scale (VAS) and neck disability index (NDI) scores, respectively, in the Tuina and TTRE groups. VAS measures pain intensity, while NDI assesses neck pain-related disability. The data demonstrate the progression or improvement in symptoms and functional outcomes across the observed period.

Secondary outcomes were reported in Table 3. The TTRE group demonstrated significantly greater improvements compared with the Tuina group in NDI (−1.5; 95% CI, −2.1 to −0.9), PSCM for extension (−2.3; 95% CI, −3.5 to −1.1), and CROM for flexion (3.7°; 95% CI, 0.3° to7.2°) at week 4. Moreover, the TTRE group demonstrated a greater reduction in VAS compared with the Tuina group at week 3 (−0.3; 95% CI, −0.52 to −0.02; *p* = 0.038). Longitudinal trends in VAS and NDI are illustrated in Figures 2A,B.

**Table 3:** Secondary outcomes by group in mechanical neck pain.

Secondary outcomes	Intervention (*n* = 45)		Control (*n* = 45)		Intervention effect	
	Mean (SD)	Mean change from baseline (95% Cl)	Mean (SD)	Mean change from baseline (95% Cl)	Difference between group (95% Cl)	*P-*value
NDI scores						
Baseline	18.20(0.4)		18.02(0.5)			
1 wk	15.98(0.4)	−2.2(−2.7 to −1.7)	16.22(0.4)	−1.8(−3.0 to −0.6)	−0.2(−1.5 to 1.0)	0.693
2 wk	12.80(0.4)	−5.4(−6.3 to −4.5)	13.16(0.5)	−4.9(−6.2 to −3.6)	−0.4(−1.6 to 0.9)	0.575
3 wk	7.04(0.3)	−11.2(−12.0 to −10.3)	8.02(0.3)	−10.0(−11.1 to −8.9)	−1.0(−1.9 to 0.1)	0.351
4 wk	3.60(0.2)	−14.6(−15.4 to −13.8)	5.13(0.2)	−12.9(−13.9 to −11.9)	−1.5(−2.1 to −0.9)	**0.001** ^*^
PSCM, lb						
Flexion						
Baseline	6.49(1.7)		6.69(2.0)			
4 wk	7.98(1.9)	−1.49(−1.9 to −1.1)	7.86(2.2)	−1.17(−1.6 to −0.7)	0.1(−1.0 to 0.7)	0.787
Extension						
Baseline	9.75(2.5)		9.18(2.8)			
4 wk	12.15(2.8)	−2.39(−3.0 to −1.8)	9.85(2.9)	−1.68(−2.2 to −1.2)	−2.3(−3.5 to −1.1)	**<0.001** ^*^
Left lateral flexion						
Baseline	8.64(2.2)		8.10(2.5)			
4 wk	9.83(2.3)	−1.20(−1.8 to −0.6)	9.42(2.7)	−1.32(−1.9 to −0.7)	−0.4(−1.4 to 0.6)	0.436
Right lateral flexion						
Baseline	8.47(2.3)		8.18(2.7)			
4 wk	9.87(2.5)	−1.40(−2.0 to −0.8)	9.75(2.8)	−1.57(−2.2 to −1.0)	−0.1(−1.2 to 1.0)	0.822
CROM, degree						
Flexion						
Baseline	53.24(7.4)		50.38(7.5)			
4 wk	57.69(7.2)	−3.80(−5.4 to −2.2)	53.96(9.2)	−5.93(−8.0 to −3.9)	−3.7(−7.2 to −0.3)	**0.035** ^*^
Extension						
Baseline	53.53(10.7)		49.82(11.0)			
4 wk	58.40(8.9)	−4.87(−7.0 to −2.7)	55.76(12.6)	−3.58(−5.2 to −1.9)	−2.6(−7.2 to 1.9)	0.253
Left lateral flexion						
Baseline	48.91(8.5)		45.78(7.7)			
4 wk	51.58(7.4)	−1.98(−3.6 to −0.4)	49.38(7.5)	−3.60(−5.0 to −2.2)	−2.2(−5.3 to 0.9)	0.164
Right lateral flexion						
Baseline	50.33(8.5)		46.87(10.1)			
4 wk	54.29(7.7)	−3.96(−5.5 to −2.5)	50.49(8.5)	−3.62(−5.3 to −1.9)	−2.8(−8.2 to 0.4)	0.290
Left rotation						
Baseline	76.69(7.5)		78.64(7.3)			
4 wk	79.91(7.9)	−3.22(−5.0 to −1.5)	81.96(8.7)	−3.31(−5.4 to −1.2)	2.0(−1.4 to 5.5)	0.247
Right rotation						
Baseline	79.67(7.2)		78.51(8.3)			
4 wk	82.69(5.7)	−3.02(−4.8 to −1.2)	80.98(7.6)	−2.47(−4.6 to −0.4)	−1.7(−4.5 to 1.1)	0.227
Cobb angle, degree						
Baseline	9.15(4.1)		9.14(4.3)			
4 wk	9.62(4.2)	−0.47(−0.6 to −0.4)	9.46(4.5)	−0.32(−0.4 to −0.2)	−0.2(−2.0 to 1.7)	0.863

Significant *p*-values are in bold font.

CROM, cervical range of motion; NDI, neck disability index; PSCM, peak strength of cervical muscles; SD, standard deviation;95% Cl, 95% confidence interval.

### 3.3 Adverse events

No adverse events were reported during the trial.

## 4 Discussion

Our findings suggest that TTRE was associated with statistically greater reductions in neck pain and functional limitations compared with Tuina alone. Both groups showed statistically significant reductions in pain and improvements in function compared with baseline after 4 weeks. However, although the between-group difference in mean VAS reduction (0.5 points) reached statistical significance, it did not exceed the established minimal clinically important difference (MCID) of 1.2–1.5 points (15, 52), indicating that the observed superiority was limited in short-term clinical relevance. The statistically significant difference in the primary outcome (pain VAS) may be partly attributable to the small variability in patient-reported pain (i.e., small SD values) in both treatment arms. This modest short-term between-group difference likely reflects real-world clinical conditions, in which Tuina itself provides appreciable analgesic effects, thereby attenuating the incremental benefit of the combined intervention. Importantly, emerging evidence suggests that clinically meaningful between-group differences may become more apparent with longer intervention durations. For instance, a recent RCT in patients with chronic neck pain reported that the MCID for pain reduction with manual therapy combined with cognitive therapy increased to 1.974 at week 8, representing a 56.17% increase compared with week 4 (53). Collectively, these findings suggest that the 4-week intervention period may have been insufficient to capture cumulative or delayed clinical benefits, underscoring the need for longer treatment durations and post-intervention follow-up in future studies.

Notably, a higher proportion of TTRE participants exceeded the MCID threshold (83.3% vs. 66.7%, *p* < 0.05), corresponding to a 16.6% absolute increase in clinically meaningful pain relief. Although the mean difference was modest, TTRE enhanced the probability of achieving meaningful individual improvement, highlighting its clinical relevance in clinical practice. Interestingly, a statistically significant between-group difference in VAS scores emerged at week 3, although the mean difference was modest (0.3 points), likely reflecting the homogeneous baseline characteristics of participants (e.g., age and occupation) that contributed to similar initial pain levels. The narrow confidence interval indicates a precise estimate. By week 4, the difference increased to 0.5 points. The progressive widening of this gap suggests a cumulative treatment effect, warranting evaluation over longer intervention periods. Notably, improvements in VAS scores preceded NDI gains by 1 week. This is clinically relevant, as individuals with neck pain primarily seek care for persistent pain and activity limitations (54, 55), and effective pain relief may reduce pain-related fear and promote functional recovery (56).

For the secondary outcomes, although statistically significant changes were observed, effect sizes were small and the confidence intervals were wide. At week 4, greater improvements were noted in NDI, flexion CROM and extension PSCM in the TTRE group. While not all variables reached statistical significance, the overall pattern suggests that incorporating RE enhances cervical muscle strength across multiple directions and improves range of motion, consistent with the protocol’s design. Previous studies (57, 58) have reported that RE can increase cervical flexor strength by 28–110% (2.6–5.1 kg) and extensor strength by 16–69% (2–8 kg). A recent study reported greater improvements in cervical flexor strength (51%, 3.26 kg) than extensor strength (33%, 1.78 kg) following a progressive shoulder-neck exercise program incorporating cranio-cervical flexion exercises targeting the extensors (59). Our RE protocol was based on the similar principle, which may explain why only extension PSCM improved significantly. Both groups demonstrated CROM increases across all directions, with the TTRE group exhibiting greater flexion CROM gains. This finding aligns with Lee et al. (60), who reported that modified cervical and shoulder retraction exercises improved cervical lordosis, potentially contributing to increased flexion CROM.

Guidelines for MNP recommend exercise-based multimodal interventions (8, 61, 62). Nonetheless, robust evidence supporting a specific multimodal approach or subtype of exercise therapy remains limited (61–63). Recent RCTs on Tuina for neck pain have provided a potential basis supporting exercise-combined therapies (15, 64). Several scholars have proposed that classifying neck pain by patient characteristics and tailoring exercise accordingly may improve outcomes (65, 66). Progressive RE served as a personalized intervention, emphasizing gradual adaptation to intensity and frequency in this trial. RE-combined therapy produced greater improvements in pain and function than Tuina alone. Incorporating social cognitive strategies (e.g., video sharing and therapist feedback via WeChat) likely enhanced adherence and engagement, contributing to the high completion rate.

Owing to the exclusion of structural pathology in MNP, Tuina is an appropriate intervention with a favorable safety profile (15). Incorporating home-based RE may reduce the need for frequent Tuina sessions, improve cost-effectiveness, and support the transition from passive care to active self-management. This RCT demonstrates that integrating Tuina with RE effectively alleviates neck pain. Positive outcomes across standardized measures (VAS, NDI, PSCM, and CROM) suggest the potential clinical value of this combined approach and provide preliminary support for its use as an evidence-based therapeutic option.

Nevertheless, several limitations should be acknowledged. First, blinding of participants and therapists was not feasible, introducing potential performance bias. Second, this single-center study involved a relatively homogeneous sample, and potential self-selection may further limit its representativeness, thereby constraining the generalizability of the findings to broader clinical settings, diverse populations, and different healthcare contexts. Third, only the immediate effects of the 4-week intervention were assessed, with no independent follow-up, leaving the durability of treatment effects and potential symptom decay or rebound unknown. While suitable for evaluating short-term responses, this design limits the applicability of the findings to real-world clinical decision-making, where sustained functional improvement is a primary goal. Finally, the individual clinical effects of RE were not assessed and should be investigated in future trials.

To address these limitations and strengthen the evidence base, future research should prioritize (1) large-scale, multicenter, and (when feasible) blinded RCTs to enhance external validity and reduce bias; (2) the incorporation of methodologically rigorous, long-term follow-up assessments (e.g., at 3, 6, and 12 months post-intervention) to evaluate the durability of effects, cost-effectiveness, and the potential for symptom rebound; and (3) well-designed studies to isolate and quantify the specific contribution of RE to the observed outcomes.

## 5 Conclusion

In this RCT, participants with MNP who received TTRE showed statistically greater improvements in pain intensity, disability, extension PSCM and flexion CROM than those who underwent Tuina therapy alone. These findings suggest that the integration of Tuina with RE can provide short-term benefits for pain and function in MNP. Therefore, the integration of Tuina with RE is recommended as an adjunctive therapeutic approach for enhancing pain relief and functional outcomes in patients with MNP. To establish its long-term efficacy, clinical sustainability, and cost-effectiveness, large-scale trials with extended follow-up periods are warranted. Future research should also aim to determine the optimal dosage and delivery format of the combined intervention for broader clinical implementation.

## Data Availability

The raw data supporting the conclusions of this article will be made available by the authors, without undue reservation.
