# Supplementary material for: Tuina therapy plus resistance exercise vs. Tuina alone for mechanical neck pain: a randomized controlled trial
Source: Front Med (Lausanne). 2026 Jan 19;13:1709232. doi: 10.3389/fmed.2026.1709232 (PMC12862936; doi:10.3389/fmed.2026.1709232)
Supplement: Supplementary file 1 [file Supplementary_file_1.pdf]

Appendix.
Tuina Protocol

Step 1: Yi Zhi Chan pushing

Operation description

The practitioner performs Yi Zhi Chan pushing manipulation along the bilateral cervical paraspinal lines. The stimulated area may gradually be expanded to regions such as the scapular back area and scapular region, with the focus progressively concentrating on "sinew nodes." This procedure lasts for 3 to 5 minutes.

Operating Area

1. **Primary Line:** Bilateral cervical paraspinal lines (vertical lines 1.5 cm lateral to C1-C7 spinous processes)
2. **Extended Area:** Scapular region (area lateral to T1-T6 spinous processes), scapular area (supraspinatus, infraspinatus, medial border of scapula)

Operational Parameters

1. **Force Control:** Initial force 2-3 kg (reference: thumb pressing creates ~1 cm indentation on test surface), adjusted to 4-6 kg based on patient feedback.
2. **Frequency:** 120-140 times/minute.
3. **Adjustment Criteria**

1) If patient reports local soreness/distension (VAS ≤3/10): Maintain current force.
2) If patient reports sharp pain (VAS ≥4/10): Immediately reduce force to 2-3 kg.
3) If palpable obvious "sinew node" (hard nodule diameter ≥0.5 cm): Increase focused manipulation at that point by 30 seconds.

4. **Quality Control:** After manipulation, local skin should be slightly reddened, and patient should report ≥20% reduction in stiffness.

Standard Duration

4 min ±30 sec

Step 2: Rolling combined with passive movements

Operation description

The practitioner applies the rolling manipulation along the neck, shoulder, and back for 2 to 3 minutes, accompanied by gentle, small-amplitude passive movements of the head and neck.

Operating Area

Cervical region (C1-T1), shoulder and upper back (upper/middle trapezius, levator scapulae surface projection).

Operational Parameters

1. **Rolling Parameters**
1) Force: 3-5 kg (vertical pressure generated by forearm rotation).
2) Frequency: 80-100 times/minute.
3) Moving Speed: 2-3 cm/sec.
2. **Passive Movement Parameters**
1) Range of Motion: Flexion/extension 20°-30°, rotation 15°-20°, lateral bending 10°-15°.
2) Movement Speed: Flexion/extension: 2 sec/cycle, rotation: 3 sec/cycle.
3) Timing: Performed during the muscle relaxation phase (exhalation) of the rolling manipulation.
3. **Adjustment Criteria**
1) If patient reports dizziness or pulling pain: Immediately reduce passive movement amplitude by 50%.
2) If significant muscle spasm is present: Pause passive movement, reduce rolling force to 2-3 kg for 30 seconds.

Standard Duration

2 min 30 sec ± 15 sec

Step 3: Pull-stretching and subtle adjusting cervical spine

Operation description

The practitioner places both thumbs on Fengchi (GB20) or the mastoid process behind the ears, with the remaining fingers supporting both sides of the mandible. Using the shoulders as a fulcrum, the practitioner raises both hands upward while shifting the body's center of gravity downward. Following the patient's breathing, the practitioner gently performs cervical traction for approximately 10 seconds. Maintaining this position, the practitioner then applies small-amplitude rotational movements to the cervical spine to subtly adjust segments affected by "bone dislocation," thereby correcting the biomechanical stress and torsion in the misaligned vertebrae.

Operational Parameters

1. **Hand Placement**
1) Thumbs: Placed on Fengchi (GB20) or posterior to mastoid process.
2) Remaining fingers: Support both sides of the mandible (avoid larynx and carotid artery).
2. **Traction Force:** 10%-15% of patient's body weight (approx. 6-12 kg), enough to create slight separation of cervical intervertebral spaces.
3. **Traction Duration:** Sustained traction for 10 sec ±2 sec.
4. **Rotation Parameters**
1) Amplitude: Left/right rotation 5°-8° each.
2) Speed: 2°-3°/sec.
3) Segment: Targeted at the "bone dislocation" segment (determined by palpation, often C3/4, C4/5).
5. **Breath Coordination:** Begin traction at end of patient's exhalation, maintain during inhalation, perform micro-adjustment during next exhalation.
6. **Adjustment Criteria**
1) History of vertebral artery type cervical spondylosis: Cancel rotation, perform vertical traction only.
2) Significant osteoporosis: Reduce traction force to 5 kg.

Standard Duration

45 sec ± 5 sec

Step 4: Pressing Tianzong (SI11)

Operation description

The practitioner applies thumb pressure technique to the patient's bilateral Tianzong points (SI 11). The thumbs are oriented perpendicular to the direction of the infraspinatus muscle fibers, approximately pointing toward the Dazhui point (GV 14). The remaining fingers are naturally placed on the patient's bilateral shoulders for stabilization, maintaining the position for approximately 15 seconds.

Operational Parameters

1. **Location:** Center of infrascapular fossa, approximately at the upper 1/3 of the line connecting the inferior border of the scapula and the medial border of the scapula.
2. **Thumb Direction:** Perpendicular to infraspinatus muscle fibers, pointing towards Dazhui (GV14).
3. **Technique Details:** Both thumbs simultaneously placed on bilateral Tianzong points, remaining fingers naturally fixed on the anterior shoulders for stabilization.
4. **Operational Force:** 4-5 kg (deep stimulation of infraspinatus, optimal sensation is soreness radiating to shoulder/arm).
5. **Operation Method:** Sustained vertical downward pressure combined with minimal kneading (amplitude ≤0.5 cm).
6. **Adjustment Criteria**

1) If patient reports severe local pain: Reduce force to 3 kg, shorten duration to 10 seconds.
2) If palpable obvious cord (infraspinatus spasm): Add 2-3 plucking motions perpendicular to muscle fibers during pressure.

Standard Duration

15 sec ± 2 sec

Step 5: Grasping Jianjing (GB21)

Operation description

The practitioner performs the grasping technique on the Jianjing point (GB21) for approximately 15 seconds, concluding the manual operation.

Operational Parameters

1. **Location:** Midpoint of the line connecting Dazhui (GV14) and the acromion, at the prominence of the upper trapezius.
2. **Technique Details:** Thumb opposes index and middle finger pulps, pinching the belly of the upper trapezius, forming a pincer shape.
3. **Operational Force:** 3-4 kg (enough to lift the trapezius belly 1-2 cm).
4. **Operational Rhythm:** One cycle is "grasp 10 sec, release 3 sec," repeated twice (total ~20 sec).
5. **Technical Requirement:** Use finger pulp force, avoid scratching with fingernails; maintain stable grip without sliding after lifting muscle.
6. **Adjustment Criteria**

1) Muscular patients: May appropriately increase force to 5 kg to ensure effective muscle lifting.
2) Thin or sensitive patients: Substitute with thumb kneading technique, force 2-3 kg, duration 15 sec.

Standard Duration

20 sec

Supplementary Note:

1. *The magnitude of force applied in each manipulation technique, as well as the corresponding frequency and duration, were determined with reference to standard Tuina textbooks used in Chinese higher education institutions and the associated literature.*
2. *A detailed description and visual demonstration of the full Tuina protocol have been published previously and are cited here for readers seeking comprehensive methodological details. (Reference: Zheng, S, Xing, H, Shan, Y, Fu, Y, Li, Y, Chen, J, et al. Chief physician SUN Wuquan's experience collection in treating neck-type cervical spondylosis with Tuina therapy. J Acupunct Tuina Sci. (2023) 21:398–404. DOI: https://doi.org/10.1007/s11726-023-1398-9.)*
