# Supplementary material for: Tuina therapy plus resistance exercise vs. Tuina alone for mechanical neck pain: a randomized controlled trial
Source: Front Med (Lausanne). 2026 Jan 19;13:1709232. doi: 10.3389/fmed.2026.1709232 (PMC12862936; doi:10.3389/fmed.2026.1709232)
Supplement: Supplementary file 2 [file Supplementary_file_2.pdf]

上海中醫藥大學附属岳阳中西医结合医院
Yueyang Hospital of Integrated Traditional Chinese and Western Medicine, Shanghai University of Traditional Chinese Medicine

Resistance Exercise Program

Each day: Do each exercise 3 sets.

First week: Repeat each set **5** times;

Second week: Repeat each set **10** times;

Third week: Repeat each set **15** times;

Fourth week: First week: Repeat each set **20** times;

Each time: Hold the maximum isometric contraction position 5s and Rest 2s.

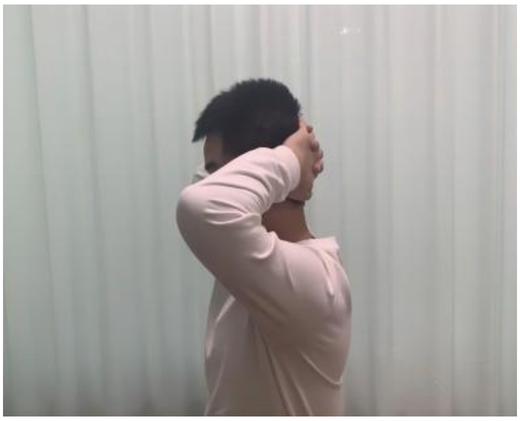

Key Points of RE

keep sitting or standing, interlock their fingers and place them above the occipital, on the back of the head, then push hands forward while simultaneously pushing head backward to generate the greatest amount of static force [1].

1. Zheng San, Xing Hua, Shan Yiming, et al. Chief physician SUN Wuquan's experience collection in treating neck-type cervical spondylosis with Tuina therapy. Journal of Acupuncture and Tuina Science, 2023, :398-404. DOI: 10.1007/S11726-023-1398-9.
